# Healthcare workers’ attitudes and practices around environmental sustainability in infection prevention

**DOI:** 10.1017/ash.2025.68

**Published:** 2025-04-15

**Authors:** Ashley L. Lin, Michelle E. Doll, Rachel J. Pryor, Elizabeth A. Monsees, Priya Nori, Gonzalo M. Bearman

**Affiliations:** 1 Department of Internal Medicine, Virginia Commonwealth University, Richmond, VA, USA; 2 Division of Infectious Diseases, Virginia Commonwealth University, Richmond, VA, USA; 3 Virginia Hospital & Healthcare Association, Richmond, VA, USA; 4 Children’s Mercy, Infection Prevention & Stewardship Integration, University of Missouri-Kansas City School of Medicine, Kansas City, MO, USA; 5 Department of Medicine, Division of Infectious Diseases, Albert Einstein College of Medicine, New York, NY, USA

## Abstract

We assessed healthcare workers’ knowledge, attitudes, and practices around disposable personal protective equipment (PPE) use. We observed that healthcare workers are interested in sustainable policies and identified areas for policy changes to reduce PPE waste.

## Introduction

The health care system is a major contributor to the environmental crisis. Climate change is linked to increased incidence of environmental-related diseases, the spread of water- and vector-borne diseases, and catastrophic weather events that directly cause casualties such as heat waves, floods, and hurricanes.^1^ In 2021, the World Health Organization published The Health Argument for Climate Action, which stated “climate change is the single biggest health threat facing humanity.”^1^ The health care system is responsible for 10% of national greenhouse gas emissions.^2^ Infection prevention protocols, including the use of disposable personal protective equipment (PPE), contribute to significant pollution.^3^ This topic is particularly relevant after the COVID-19 pandemic, which led to an increase in PPE use, particularly single-use PPE.^4,5^ We assessed healthcare workers’ knowledge and attitudes towards environmental sustainability and infection prevention in the healthcare setting.

## Methods

Approximately 5000 staff physicians, advanced practice providers (APPs), and inpatient and emergency department (ED) nurses at Virginia Commonwealth University Health System (VCUHS) were sent a voluntary RedCap survey of 15 questions rated on a Likert scale from “strongly agree” to “strongly disagree.” Questions were asked about topics including respondents’ motivations for using PPE (eg, for infection prevention, self-protection, or due to hospital policy), PPE use in certain situations (eg, for brief encounters, non-essential encounters, in COVID-19 patients’ rooms, or in rooms of patients with historical multi-drug resistant (MDR) infections), and attitudes and knowledge around PPE use and sustainability (eg, whether they believe the benefits of PPE use outweigh the environmental impacts, whether they have received adequate education on sustainability in healthcare). Descriptive statistics were performed using Microsoft Excel. “Strongly agree” and “agree” were grouped together as “agree,” and “strongly disagree” and “disagree” were grouped together as “disagree.” Not all respondents answered all questions, so unanswered questions were excluded from the subgroup analysis.

## Results

Four hundred sixty-five survey responses were analyzed, with a 9.3% response rate, including responses from 109 physicians, 43 APPs, and 305 nurses working in a variety of settings (Table 1). Most respondents agreed with more than one motivation for PPE use, such as hospital policy (87% (401/463)) or self-protection (77% (354/461)). Forty-nine percent (227/462) of survey respondents agreed that sterilized reusable PPE is safe compared to disposable PPE. Fifty-four percent (252/463) of all respondents agreed that the benefits of PPE use outweigh the environmental impacts of PPE. Two-thirds of all respondents agreed they minimize non-essential encounters for patients with isolation precautions. A minority of respondents (24% (109/460)) were sufficiently aware of environmental sustainability in healthcare. Only 14% (65/459) agreed that the healthcare workplace promotes environmental sustainability. Survey results are summarized in Table 2.

**Table 1.** Respondent demographics

	Number of respondents (% of total respondents)
What is your role?	
Fellow physician	1 (0.2%)
Physician associate	11 (2.4%)
Nurse practitioner	32 (7.0%)
Attending physician	108 (23.6%)
Registered nurse	305 (66.7%)
What setting do you primarily work in?	
Emergency Department	31 (6.8%)
Interventional Radiology/Procedure Unit	38 (8.3%)
ICU	93 (20.3%)
Outpatient clinic	138 (30.1%)
Non-ICU inpatient	158 (34.5%)
How many years have you been in practice (including residency and fellowship)?	
1-2	32 (6.9%)
3–5	42 (9.1%)
6–10	88 (19.1%)
11–15	73 (15.8%)
16–20	76 (16.5%)
>21	150 (32.5%)

**Table 2.** Survey results for all respondents

Question	Strongly Agree/Agree	Neither agree nor disagree	Strongly disagree/disagree
1. The benefits of personal protective equipment (PPE) use outweigh the environmental impacts of PPE	252/463 (54%)	124/463 (27%)	87/463 (19%)
2. I have received sufficient education about environmental sustainability in healthcare.	109/460 (24%)	55/460 (12%)	296/460 (64%)
3. I am aware of the indications for PPE use.	446/460 (97%)	8/460 (2%)	6/460 (1%)
4. I use disposable PPE because I believe it is necessary for infection prevention.	361/461 (78%)	59/461 (13%)	41/461 (9%)
5. I use disposable PPE because I believe it is necessary for self-protection.	354/461 (77%)	69/461 (15%)	38/461 (8%)
6. I use disposable PPE because it is required by hospital policy.	401/463 (87%)	45/463 (10%)	17/463 (4%)
7. I believe sterilized reusable PPE is a safe option when compared to disposable PPE.	227/462 (49%)	164/462 (35%)	71/462 (15%)
8. I always use PPE when required, no matter how brief the encounter.	317/461 (69%)	59/461 (13%)	85/461 (18%)
9. My team minimizes non-essential encounters for patients with isolation precautions.	301/455 (66%)	97/455 (21%)	57/455 (13%)
10. I use gloves for all inpatient encounters.	267/463 (58%)	53/463 (11%)	143/463 (31%)
11. I use gowns in COVID patients’ rooms because I believe it reduces transmission.	227/461 (49%)	119/461 (26%)	115/461 (25%)
12. I use gowns in COVID patients’ rooms because it is hospital policy.	377/459 (82%)	56/459 (12%)	26/459 (6%)
13. I use gowns in the rooms of patients with historical multi-drug resistant (MDR) infections because I believe it reduces transmission.	276/460 (60%)	114/460 (25%)	70/460 (15%)
14. I use gowns in the rooms of patients with historical MDR infections because it is hospital policy.	371/459 (81%)	64/459 (14%)	24/459 (5%)
15. My workplace has programs in place to increase environmental sustainability.	65/459 (14%)	144/459 (31%)	250/459 (54%)

Respondents’ role and setting also influenced their clinical practice around PPE. Sixty-seven percent (82/107) of physicians agreed that sterilized reusable PPE is safe compared to disposable PPE, but only 44% (133/302) of nurses agreed. Eighty-one percent (109/134) of outpatient provider respondents agreed they always use PPE no matter how brief the encounter, compared to only 42% (13/31) of those working in the ED. Forty-nine percent (227/461) of respondents agreed that they wear gowns in COVID-19 patients’ rooms because they believe it reduces transmission. In contrast, 82% (377/459) of respondents agreed that they wear gowns in COVID-19 patients’ rooms due to hospital policy. ED respondents were the least likely to agree (only 16% (5/31) agreement) that they wear gowns in COVID-19 patients’ rooms because they believe it reduces transmission.

## Discussion

The determination of precautions for infection prevention comes from national guidelines, professional society position papers, and recommendations from groups such as the Healthcare Infection Control Practices Advisory Committee, which lag current research. The results highlight opportunities to optimize environmentally sustainable strategies for PPE use at VCUHS. Scant evidence supports COVID-19 transmission via clothing yet gowning in COVID-19 patients’ rooms remains hospital policy.^6^ The finding that less than half of respondents believe gowns reduce COVID-19 transmission, which is also supported by literature, suggests that a policy change ending mandatory gowning in COVID-19 patients’ rooms would be well accepted. Another potential policy change is minimizing non-essential encounters for patients with isolation precautions, such as limiting the team members entering an isolation room to only those directly caring for the patient.

We found that healthcare workers may need more education on the environmental impacts of health care. Research suggests that the use of reusable items leads to reduced pollution without increasing infection rates,^7^ but less than half of respondents agreed that sterilized reusable PPE is safe compared to disposable PPE. Most respondents disagreed that the healthcare workplace promotes environmental sustainability. This is not unique to our facility. Inadequate hospital infrastructure and lack of management support have been identified as barriers in prior studies.^8,9^

Twenty-eight percent (35/124) of respondents working in high acuity settings (ICU and ED) disagreed or strongly disagreed that the benefits of PPE use outweigh the environmental impacts, compared to 9% (13/138) of outpatient respondents. This runs counter to the theory that individuals are more likely to don PPE when they perceive a personal risk, as those working in high-acuity settings encounter infections more often than those in outpatient settings. A limitation is that respondents were not asked what they perceived to be the environmental impacts, so their response may be influenced by how much they know about sustainability.

This study elucidates VCUHS staff perceptions related to PPE practices, and this study may serve to promote discussion and further development at VCUHS. However, external validity is limited due to the relatively small sample size and the single-center study design. Additionally, resident physicians and fellows were not surveyed. Although the survey was anonymous, respondents may not have provided truthful answers either due to recall bias or out of a desire to provide the most socially acceptable answer. Respondents also received heterogeneous training on PPE policy. A distinction was not made between sterile PPE used in procedures and non-sterile PPE used in isolation precautions.

This study is the first to measure attitudes and practices around PPE use as it relates to environmental sustainability. Our study found mixed practices on the use of gowns by discipline and setting and mixed views on whether the benefit of PPE outweighed their impact on the environment. Thoughtful workflow may aid in judicious PPE use. Future directions may include ending the policy of gowning in COVID-19 patient rooms. Sustainability in healthcare remains a still largely untapped frontier for health promotion.

## Supporting information

Lin et al. supplementary material Lin et al. supplementary material

## Supplementary material

For supplementary material accompanying this paper visit http://doi.org/10.1017/ash.2025.68. click here to view supplementary material
