# Supplementary material for: Healthcare workers’ attitudes and practices around environmental sustainability in infection prevention
Source: Antimicrob Steward Healthc Epidemiol. 2025 Apr 15;5(1):e94. doi: 10.1017/ash.2025.68 (PMC12022923; doi:10.1017/ash.2025.68)
Supplement: Lin et al. supplementary material [file S2732494X25000683sup001.pdf]

Environmental Sustainability survey

Thank you for helping us understand the attitudes around environmental sustainability and personal protective equipment (PPE) use at VCUHS.

1) What is your role?
- ◯ Registered nurse
- ◯ Nurse practitioner
- ◯ Physician associate
- ◯ Resident physician
- ◯ Fellow physician
- ◯ Attending physician

2) What setting do you primary work in?
- ◯ ICU
- ◯ Non-ICU inpatient
- ◯ Emergency Department
- ◯ Interventional Radiology/Procedure Unit
- ◯ Outpatient clinic

3) How many years have you been in practice (including residency and fellowship)?
- ◯ 1-2
- ◯ 3-5
- ◯ 6-10
- ◯ 11-15
- ◯ 16-20
- ◯ >21

4) The benefits of personal protective equipment (PPE) use outweight the environmental impacts of PPE
- ◯ Strongly disagree
- ◯ Disagree
- ◯ Neither agree nor disagree
- ◯ Agree
- ◯ Strongly agree

5) I have received sufficient education about environmental sustainability in healthcare. Specifically, I understand how the healthcare system contributes to the current environmental crisis and the negative downstream health effects of this crisis.
- ◯ Strongly disagree
- ◯ Disagree
- ◯ Neither agree nor disagree
- ◯ Agree
- ◯ Strongly agree

6) I am aware of the indications for PPE use.
- ◯ Strongly disagree
- ◯ Disagree
- ◯ Neither agree nor disagree
- ◯ Agree
- ◯ Strongly agree

7) In general, I use disposable PPE because I believe it is necessary for infection prevention.
- ◯ Strongly disagree
- ◯ Disagree
- ◯ Neither agree nor disagree
- ◯ Agree
- ◯ Strongly agree

8) In general, I use disposable PPE because I believe it is necessary for self-protection.
- ◯ Strongly disagree
- ◯ Disagree
- ◯ Neither agree nor disagree
- ◯ Agree
- ◯ Strongly agree

9) In general, I use disposable PPE because it is required by hospital policy.
- ◯ Strongly disagree
- ◯ Disagree
- ◯ Neither agree nor disagree
- ◯ Agree
- ◯ Strongly agree

10) I believe sterilized reusable PPE is a safe option when compared to disposable PPE.

- ◯ Strongly disagree
- ◯ Disagree
- ◯ Neither agree nor disagree
- ◯ Agree
- ◯ Strongly agree

11) I always use PPE when required, no matter how brief the encounter.

- ◯ Strongly disagree
- ◯ Disagree
- ◯ Neither agree nor disagree
- ◯ Agree
- ◯ Strongly agree

12) My team minimizes non-essential encounters for patients with isolation precautions (for example, team members not directly taking care of the patient would not enter the room on rounds unless there is a learning opportunity from seeing the patient).

- ◯ Strongly disagree
- ◯ Disagree
- ◯ Neither agree nor disagree
- ◯ Agree
- ◯ Strongly agree

13) I use gloves for all inpatient encounters.

- ◯ Strongly disagree
- ◯ Disagree
- ◯ Neither agree nor disagree
- ◯ Agree
- ◯ Strongly agree

14) I use gowns in COVID patients' rooms because I believe it reduces transmission.

- ◯ Strongly disagree
- ◯ Disagree
- ◯ Neither agree nor disagree
- ◯ Agree
- ◯ Strongly agree

15) I use gowns in COVID patients' rooms because it is hospital policy.

- ◯ Strongly disagree
- ◯ Disagree
- ◯ Neither agree nor disagree
- ◯ Agree
- ◯ Strongly agree

16) I use gowns in the rooms of patients with historical multi-drug resistant (MDR) infections because I believe it reduces transmission.

- ◯ Strongly disagree
- ◯ Disagree
- ◯ Neither agree nor disagree
- ◯ Agree
- ◯ Strongly agree

17) I use gowns in the rooms of patients with historical MDR infections because it is hospital policy.

- ◯ Strongly disagree
- ◯ Disagree
- ◯ Neither agree nor disagree
- ◯ Agree
- ◯ Strongly agree

18) My workplace has programs in place to increase environmental sustainability.

- ◯ Strongly disagree
- ◯ Disagree
- ◯ Neither agree nor disagree
- ◯ Agree
- ◯ Strongly agree
